# On the anniversary of Professor, Dr. Sci. Valentina G. Kuznetsova, Editor-in-Chief of the journal “*Comparative Cytogenetics*”

**DOI:** 10.3897/compcytogen.20.182675

**Published:** 2026-01-22

**Authors:** Natalia V. Golub, Vladimir A. Lukhtanov, Natalia S. Khabazova, Boris A. Anokhin, Nazar A. Shapoval, Galina N. Shapoval, Ilya A. Gavrilov-Zimin

**Affiliations:** 1 Department of Karyosystematics, Zoological Institute, Russian Academy of Sciences, Universitetskaya emb. 1, 199034 St. Petersburg, Russia Russian Academy of Sciences Moscow Russia; 2 S.I. Vavilov Institute for the History of Science and Technology, Russian Academy of Sciences, Moscow, Russia Russian Academy of Sciences St. Petersburg Russia

**Keywords:** Bibliography, biography, biology, classical and molecular cytogenetics, evolution, Insecta, holokinetic chromosomes, karyosystematics

## Abstract

The article is dedicated to the anniversary of Professor, Dr. Sci. Valentina G. Kuznetsova, Editor-in-Chief and co-founder of the journal “Comparative Cytogenetics”. V. Kuznetsova is the principal researcher at the Zoological Institute of the Russian Academy of Sciences, Head of the Department of Karyosystematics. Under her leadership, many Russian and foreign cytogeneticists have successfully defended their dissertations. Today, Valentina Kuznetsova is one of the world’s leading experts in the field of comparative cytogenetics and karyosystematics of insects. The list of her publications includes 200 scientific articles, chapters in books and collective monographs.

On January 29, 2026, Valentina G. Kuznetsova, Professor, Doctor of Biological Sciences, Principal Researcher at the Zoological Institute of the Russian Academy of Sciences, Head of the Department of Karyosystematics at the Laboratory of Insect Systematics, Editor-in-Chief and co-founder of the journal “*Comparative Cytogenetics”*, turns 85 years old.

Valentina Kuznetsova (Fig. 1) is one of the world’s leading experts in the field of insect cytogenetics and karyosystematics. She was born in Vladivostok (Far East of Russia) in the family of a scientist-biologist and a military engineer. In 1946, the family returned to their native Leningrad (now Saint Petersburg), where Valentina enrolled in a school, from which she graduated with a silver medal. In 1958, she entered the Biology and Soil Science Faculty of Leningrad State University (now St. Petersburg State University), where she specialized in the Department of Genetics and Breeding. After graduating from the University in 1965, Valentina became a postgraduate student at the Zoological Institute of the Russian Academy of Sciences (ZIN RAS). At ZIN, her scientific supervisors were prominent scientists, Prof. Lidia A. Chubareva (1921–2006), one of the world leaders in the field of insect cytotaxonomy (specifically, the family Simuliidae, Diptera), Head of the Laboratory of Population Genetics and Karyosystematics, and Prof. Georgy Kh. Shaposhnikov (1915–1997), a well-known expert on aphid taxonomy (Figs 2, 3). Valentina was always proud to have been their student. In May 1969, she successfully defended her PhD dissertation on “Aphid karyology and possible ways of using it in the study of their evolution, phylogeny and systematics”.

**Figure 1.**
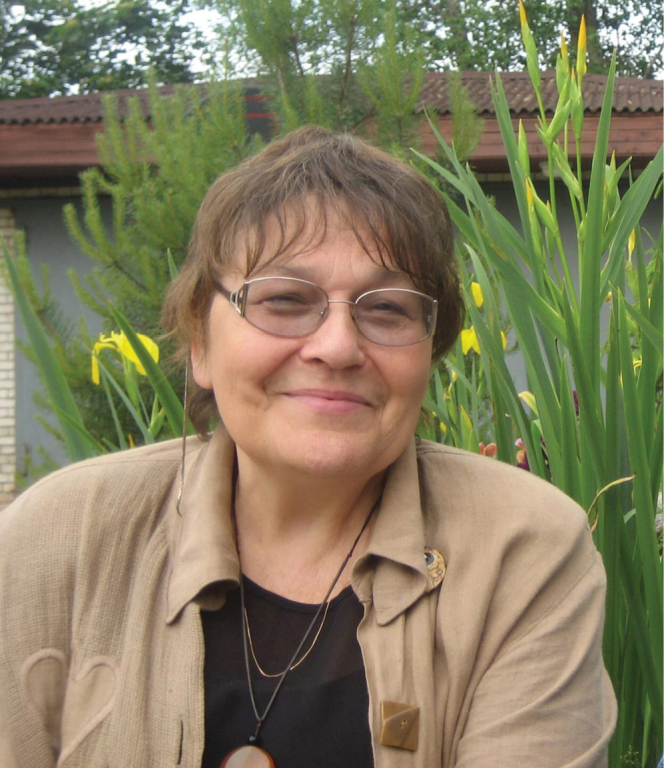
Valentina Kuznetsova.

**Figure 2.**
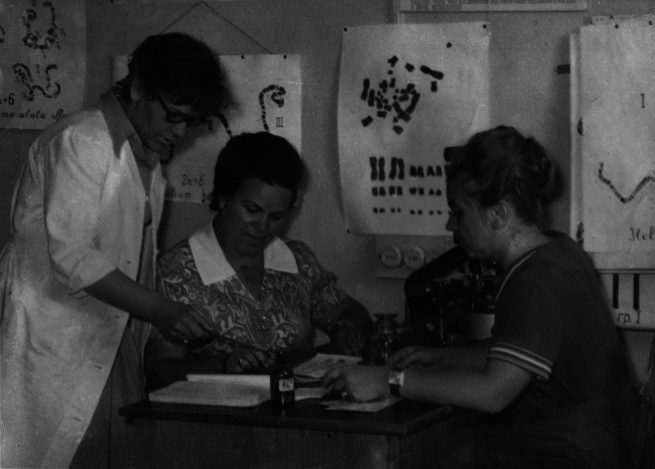
Valentina Kuznetsova during her postgraduate studies at the Zoological Institute (from left to right: Valentina Kuznetsova with her scientific supervisor Lidia A. Chubareva and Ninel Petrova).

**Figure 3.**
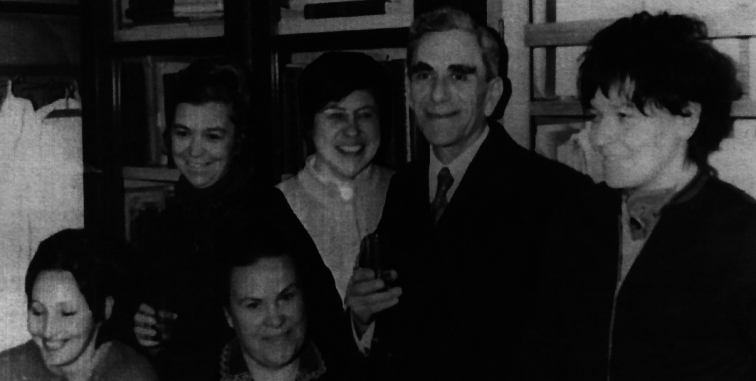
Valentina Kuznetsova during her postgraduate studies at the Zoological Institute (from left to right: Larisa Kupriyanova, Ninel Petrova, Lidia A. Chubareva, Diana Kolesova, Georgy Kh. Shaposhnikov and Valentina Kuznetsova).

Since then, Valentina’s entire scientific life has been inextricably linked with the Zoological Institute, where she rose from a junior researcher to a principal researcher (Fig. 4). In 1986, she became the Head of the Department of Karyosystematics of the Laboratory of Insect Systematics, replacing Lidia A. Chubareva, and directs the work of this Department to the present day (Fig. 5). In 1992, V. Kuznetsova defended her thesis for the degree of Doctor of Biological Sciences on the topic “Holokinetic chromosomes of insects, their evolution and taxonomic significance”.

**Figure 4.**
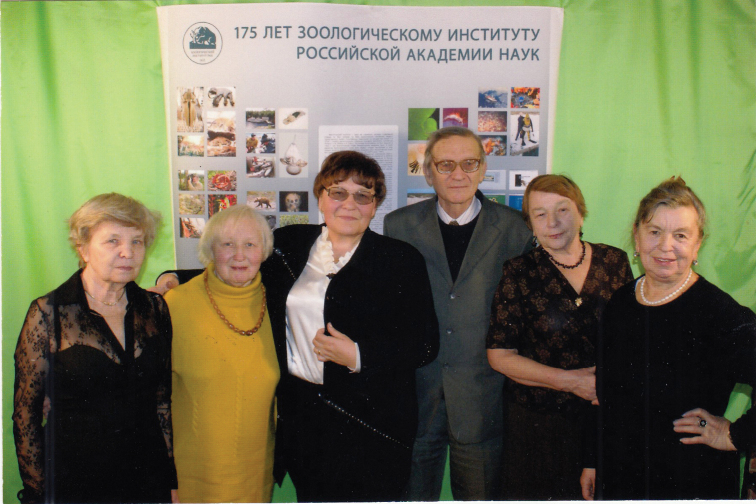
Valentina Kuznetsova with her colleagues at the 175 years anniversary of Zoological Institute of RAS (2007).

**Figure 5.**
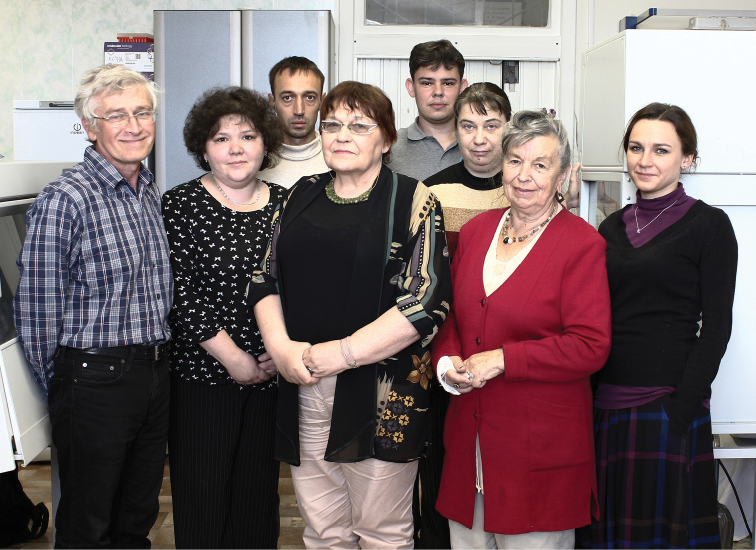
Valentina Kuznetsova with the staff of the Department of Karyosystematics in 2019 (from left to right: Vladimir Lukhtanov, Natalia Golub, Boris Anokhin, Nazar Shapoval, Natalia Khabazova, Ninel Petrova, Alisa Vershinina).

Valentina Kuznetsova’s long-term scientific activity is devoted to the study of cytogenetics and, in parallel, the structure of the testes and ovaries (which are the crucial tissues for studying chromosome structure and behavior in cell cycles), the systematics and evolution of various insect orders such as Odonata, Dermaptera, Notoptera (Mantophasmatodea), Psocoptera, Hemiptera, Lepidoptera, Neuroptera, Coleoptera, Hymenoptera, and also some other invertebrates, e.g., Cnidaria, Turbellaria and Mollusca. She pays special attention to the study of insects with holokinetic chromosomes (which lack a localized centromere) using modern methods of classical and molecular cytogenetics.

Prof. V. Kuznetsova is the author and co-author of 200 scientific articles, chapters in books and collective monographs (see the List of her publications below). In collaboration with her students and colleagues, she has written a number of theoretical papers and reviews on the structure and evolution of holokinetic chromosomes, as well as on general issues of cytogenetics, molecular systematics and phylogeography of insects, including in recent years (e.g., Gokhman & Kuznetsova 2006, 2017, 2022, 2024; Lukhtanov & Kuznetsova 2010; Kuznetsova & Grozeva 2010; Kuznetsova & Aguin-Pombo 2015; Lukhtanov….& Kuznetsova 2015; Vershinina & Kuznetsova 2016; Kuznetsova et al. 2020, 2025; Shapoval… & Kuznetsova 2021, 2025; Stoianova…& Kuznetsova 2025; Golub….. & Kuznetsova 2025 etc).

For many years, Prof. Kuznetsova has been providing extensive scientific advice and supervising the dissertation research of domestic and foreign graduate and doctoral students (Fig. 6). Ten candidate (= PhD) and five doctoral (= habilitation) dissertations were prepared and successfully defended under her scientific supervision.

**Figure 6.**
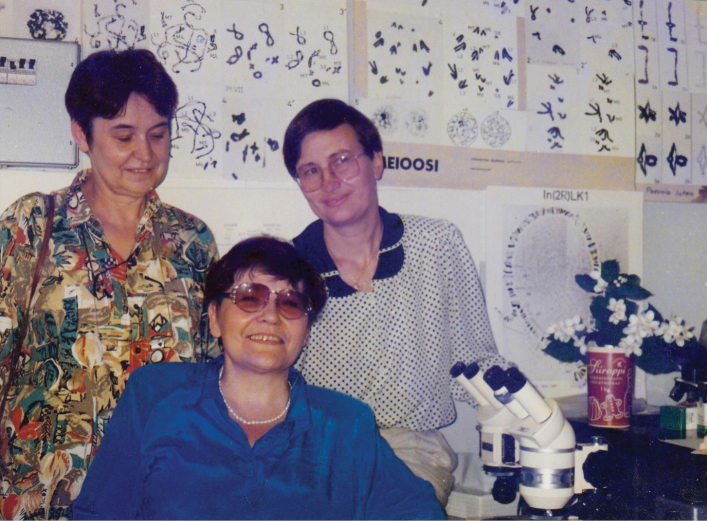
Valentina Kuznetsova with her doctoral students, Anna Maryańska-Nadachowska (Institute of Systematics and Evolution of Animals PAS, Kraków, Poland) and Snejana Grozeva (Institute of Biodiversity and Ecosystem Research BAS, Sofia, Bulgaria).

Valentina is not only a “desk scientist”. Over the years, she has actively participated in expeditions organized by the Zoological Institute to the Russian Far East, the Caucasus, Central Asia, Kazakhstan, Vietnam, and so on. She collected insects for cytogenetic research in Italy, Greece, Finland, Portugal (in particular, on the island of Madeira), Germany, Poland and Bulgaria (Fig. 7). She has worked with many colleagues in cytogenetic laboratories in various countries around the world, including Israel, Portugal, Hungary, France, Germany, Finland, Poland, Bulgaria, Georgia etc.

**Figure 7.**
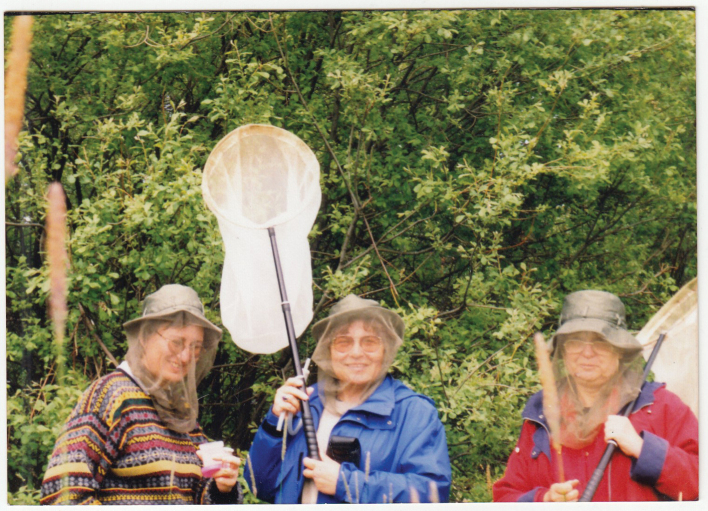
Valentina Kuznetsova collects insects with Snejana Grozeva (left) and Anna Maryańska-Nadachowska (right).

Along with scientific studies, Prof. Kuznetsova performs many organizational activities. For many years, she has been an expert in various scientific societies, such as, for example, the Russian Foundation for Basic Research (RFBR), The Russian Science Foundation (RSF), and the Federal Register of specialists in scientific and technical fields of education and science of the Russian Federation. She is currently a member of the Council of the St. Petersburg branch of the All-Russian Genetic Society and the Presidium of the Russian Entomological Society (Fig. 8). At ZIN, she is a member of the Postgraduate Studies Committee, and the Editorial and Publishing Council. She is the Editor-in-Chief and co-founder of “Comparative Cytogenetics” (together with another Editor-in-Chief of the journal, her former postgraduate student, now PhD, Dr. Sci. Ilya A. Gavrilov-Zimin). The journal has existed since 2007 and is indexed in the Web of Science and Scopus, which are the world’s leading abstract and citation databases for peer-reviewed literature. Besides, Prof. Kuznetsova is a member of the Editorial Boards of some other international genetic and zoological journals, e.g., “Insects”, “Genes” and “Folia Biologica (Kraków)”. Over the years of her work at the institute, she has been awarded many diplomas and titles; for example, in 2018 she was awarded the title of Honored Scientist of the Russian Federation.

**Figure 8.**
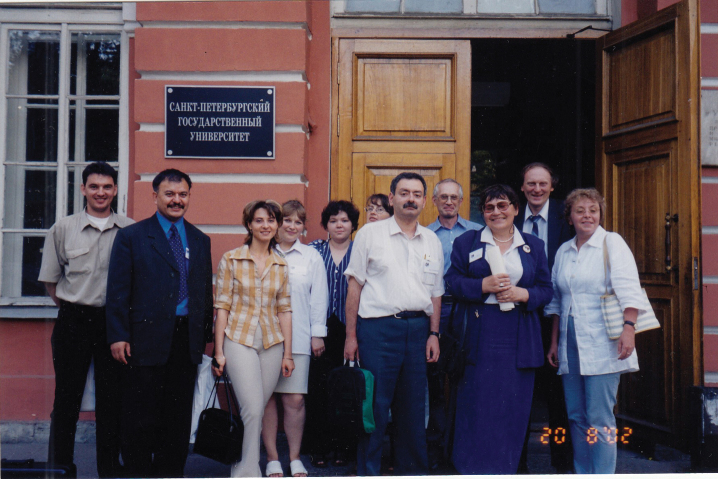
Valentina Kuznetsova with her colleagues during the XII Congress of the Russian Entomological Society.

However, the life of Valentina is not restricted only to science and administrative duties. She is an incredibly versatile person, keenly interested in literature and music, well informed and deeply understanding of them. Valentina has raised two wonderful sons, both of them have academic degrees, one became a famous zoologist and lecturer at St. Petersburg State University, and the other became a medical doctor and lecturer at one of the leading medical Universities in St. Petersburg. Valentina supports her sons and their families by participating in the upbringing of her grandchildren and great-grandchildren. Despite her incredible preoccupation with scientific and administrative matters, Valentina finds time for interesting trips, visits to natural and architectural monuments of the Leningrad Region, Karelia, and the northern and northwestern regions of Russia (Fig. 9). Incredible energy, inexhaustible interest in the world and openness to everything new fascinate everyone in her environment. Everyone who knows her emphasizes the fact that, under all circumstances, Valentina remains a deeply decent, sincere and friendly person, being an example of a true intellectual scientist.

**Figure 9.**
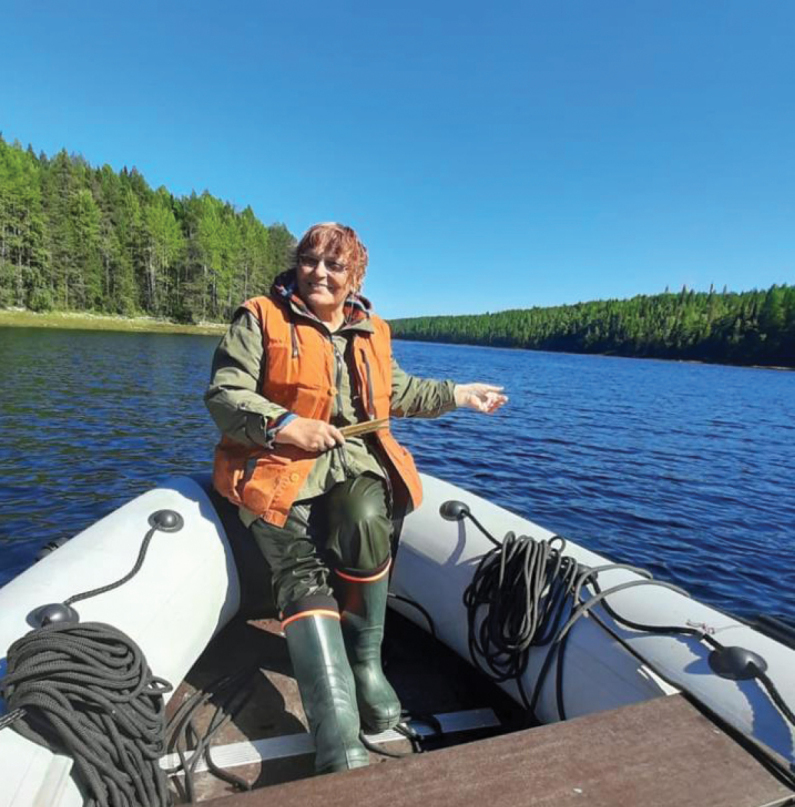
Valentina Kuznetsova fishes in the White Sea (June, 2025).

Friends and colleagues of Professor, Dr. Sci. Valentina Kuznetsova cordially congratulate her on a wonderful anniversary and wish her many more years of fruitful scientific activity and new achievements in the field of cytogenetics and karyosystematics, good health and good luck in everything.

## List of publications by Valentina G. Kuznetsova

1. Kuznetsova VG, Polyakova TF (1966) Sex chromatin in *Rumex acetosa* L. Genetika 9: 164–169. [In Russian].
2. Kuznetsova VG (1968) Karyotypes of the aphids of subtribe Anuraphidina (Aphididae) and possible ways of their evolution. Entomologicheskoe Obozrenie 47(4): 767–781. [In Russian with English summary].
3. Kuznetsova VG (1969) On the chromosome number in *Myzodes persicae* Sulz. (Homoptera, Aphididae). Tsitologia 11(3): 386–388. [In Russian with English summary].
4. Kuznetsova VG (1969) Karyology of aphids and its possible applications in studying their evolution, phylogeny, and systematics. Abstract for Ph.D. Dissertation, Leningrad, USSR: Zoological Institute, Russian Academy of Sciences, 19 pp. [In Russian].
5. Kuznetsova VG, Shaposhnikov GKh (1973) The chromosome numbers of the aphids (Homoptera, Aphidinea) of the world fauna. Entomological Review 52: 78–96.
6. Kuznetsova VG (1974) The peculiarities of the aphids chromosomes (Homoptera, Aphidinea. Tsitologia 41(7): 803–809. [In Russian with English summary].
7. Kuznetsova VG (1975) A study of related species of Aphididae with different karyotypes using DNA cytophotometry. Doklady Akademii nauk SSSR 224(2): 457–459. [In Russian].
8. Kuznetsova VG, Petropavlovskaya MB (1976) The behaviour of holokinetic chromosomes in the spermatogenesis of bugs (Hemiptera: Pentatomidae). Tsitologia 18(6): 702–711. [In Russian with English summary].
9. Kuznetsova VG (1978) The disturbance of anaphase chromosome disjunction in the cleavage of *Cavariella* sp. (Homoptera, Aphidinea). Tsitologia 20(6): 705–707. [In Russian with English summary].
10. Kuznetsova VG (1979) The chromosomes of the holokinetic type and their distribution among insects and other invertebrate animals. In: Chubareva LA (Ed.) Karyosystematics of the invertebrate animals. Leningrad, 5–19. [In Russian].
11. Kuznetsova VG (1979) The karyotype of *Labidura riparia* in West Sibiria (type-locality) and the systematics of the genus *Labidura* Leach (Dermaptera, Labiduridae). Entomological Review 58(1): 49–52.
12. Kuznetsova VG, Daniyarova MM (1980) Karyotypes of the aphids belonging to the genus *Dysaphis* Borner (Homoptera, Aphididae, Anuraphidina) from Tadzhikistan. Doklady AN Tadzhikskoy SSR 23(12): 734–736. [In Russian].
13. Kolesova DA, Kuznetsova VG, Shaposhnikov GKh (1980) Clonal variability in *Myzus persicae* Sulz. (Homoptera, Aphididae). Entomological Review 59(3): 21–34.
14. Kuznetsova VG (1980) The way of karyotype evolution in insects with holokinetic chromosomes. In: Sakai S (Ed) Some guide signs on insect integrated taxonomy. 16-th International Congress Entomology, Japan, Kyoto, 85–89, A, B.
15. Kuznetsova VG (1982) Chromosome studies of leafhoppers of the family Delphacidae (Homoptera, Auchenorrhyncha). Entomological Review 61(2): 17–23.
16. Emelyanov AF, Kuznetsova VG (1983) The number of seminal follicles as a phylogenetic and taxonomic feature in the Dictyopharidae (Homoptera) and other leafhoppers. Zoologicheskii Zhurnal 62(10): 1583–1586. [In Russian with English summary].
17. Shaposhnikov GKh, Kuznetsova VG (1984) The first inter-republican aphidological symposium “Systematics and ecology of aphids injurious to plants”. Entomologicheskoe Obozrenie 63(1): 216–218. [In Russian].
18. Kuznetsova VG, Sapunov VB (1985) The influence of X-rays on morphological and karyotypical variability of *Aphis craccivora* Koch. Tsitologia i Genetika 19(5): 387–391. [In Russian with English summary].
19. Kuznetsova VG (1986) Phylogenetic analysis of chromosome variability and karyosystematics of Cicadina of the family Dictyopharidae (Homoptera, Auchenorrhyncha). Entomological Review 65(2): 88–106.
20. Kirillova VI, Kuznetsova VG (1986) New data on the karyotypes of the family Delphacidae (Homoptera: Cicadinea). Tsitologia 28(8): 870–875. [In Russian with English summary].
21. Kuznetsova VG, Sapunov VB (1987) Effect of X-rays on the morphological and karyological inconstancy of aphids. In: Population structure, genetics and taxonomy of aphids and thysanopterans. SPB Academic Publishing, Hague, The Netherlands, 134–138.
22. Sapunov VB, Kuznetsova VG (1987) The use of aphids for genetical investigations: the state and perspective. In: Population structure, genetics and taxonomy of aphids and thysanopterans. SPB Academic Publishing, Hague, The Netherlands, 139–144.
23. Kuznetsova VG (1988) Unusual mechanism of chromosome sex determination in cotton lace bug *Dysdercus superstitiosus* F. (Pyrrhocoridae, Heteroptera). Doklady Akademii nauk SSSR 301(2): 456–458. [In Russian].
24. Kuznetsova VG, Lukhtanov VA, Kirillova VI, Koroleva YuI (1988) Karyotypes of the insects – pests of the agricultural plants. Tsitologia i genetika 22(4): 56–60. [In Russian with English summary].
25. Grozeva SM, Kuznetsova VG (1988) Karyotypes, testis follicle and ovariole numbers in the subfamily Artheneinae (Heteroptera, Pentatomomorpha, Lygaeidae). Proceedings of the XII International Symposium on Entomofauna of Central Europe. Kiev, 289–295.
26. Lukhtanov VA, Kuznetsova VG (1989) Karyotype structure in higher Lepidoptera (Papilionomorpha). Entomological Review 68(5): 12–31.
27. Lukhtanov VA, Kuznetsova VG (1989) The analysis of the karyotype variability in the butterflies of the *Melitaea didyma* group along with the evidence of the species distinctness of *M. latonigena* (Lepidoptera, Nymphalidae). Zoologicheskii Zhurnal 68(12): 38–46. [In Russian with English summary].
28. Kuznetsova VG (1989) The chromosome mechanisms of sex determination in Insecta (with reference to Hemiptera). In: Tonner M, Soldan T, Bennettova B (Eds) Regulation of Insect Reproduction IV. Proceedings of Symposium. Praha, 293–301.
29. Kuznetsova VG, Narchuk EP (1989) Insects inhabiting common ragweed in the coastal area of the Black Sea in the Caucasus. In: Kovalev OV, Belokobylskij SA (Eds) Theoretical principles of biological control of the common ragweed. Leningrad, Nauka, 224–226. [In Russian with English summary].
30. Grozeva S, Kuznetsova VG (1990) Karyotypes and some structural properties of the reproductive system of bugs of the subfamily Artheneinae (Heteroptera, Pentatomomorpha, Lygaeidae). Entomological Review 69(4): 14–26.
31. Kirillova VI, Kuznetsova VG (1990) B-chromosomes of *Javesella pellucida* Fabr. and other Delphacidae (Homoptera, Cicadinea). Tsitologia 32(3): 282–290. [In Russian with English summary].
32. Kuznetsova VG, Kirillova VI (1990) Karyotypes and anatomic characteristics of the sex system in the Fulgoroidea (Homoptera, Cicadinea). Zoologicheskii Zhurnal 69(3): 24–31. [In Russian with English summary].
33. Kuznetsova VG, Gandrabur SI (1991) The nucleolar organizing regions in the aphid chromosomes. Tsitologia 33(2): 41–47. [In Russian with English summary].
34. Kuznetsova VG (1992) Holokinetic chromosomes in Insecta, their evolution and taxonomical significance. Dr. Sc. Thesis. St. Petersburg, Russian Federation: Zoological Institute, Russian Academy of Sciences 50 pp. [In Russian].
35. Maryańska-Nadachowska A, Kuznetsova VG, Warchałowska-Śliwa E (1992) Karyotypes of Psyllina (Homoptera) I. New data and check-list. Folia biologica (Kraków) 40(1–2): 15–25.
36. Maryańska-Nadachowska A, Warchałowska-Śliwa E, Kuznetsova VG (1992) The NOR and nucleolus in the spermatogenesis of *Psylla alni* (L) (Homoptera) analysed by silver staining. Folia biologica (Kraków) 40(1–2): 41–45.
37. Grozeva S, Kuznetsova V (1992) The reproductive system of some bug families (Heteroptera, Pentatomomorpha). In: Bennettová B, Gelbič I, Soldán T (Eds) Advances in Regulation of Insect Reproduction. Institute of Entomology, Czech Academy of Sciences, 97–102.
38. Grozeva SM, Kuznetsova VG (1993) Karyotype variability in the primitive families of the bug infraorder Pentatomomorpha (Heteroptera). In: Chubareva LA, Kuznetsova VG (Eds) Karyosystematics of the invertebrate animals II. St. Petersburg, Zoological Institute RAS, 10–12. [In Russian with English summary].
39. Kuznetsova VG, Maryańska-Nadachowska A (1993) C-heterochromatin and NOR’s in X-chromosomes of aphid *Dysaphis anthrisci* Born. In: Chubareva LA, Kuznetsova VG (Eds) Karyosystematics of the invertebrate animals II. St. Petersburg, Zoological Institute RAS, 16–18. [In Russian with English summary].
40. Kuznetsova VG, Kirillova VI (1993) Karyotype and male internal reproductive system of *Aetalion reticulatum* L. (Aetalionidae, Cicadelloidea, Cicadina): phylogenetic aspect. Beiträge zur Entomologie 43(1): 119–122.
41. Grozeva SM, Kuznetsova VG (1993) Notes on the karyotypes of some lygaeid bugs (Heteroptera, Pentatomomorpha, Lygaeidae). Folia biologica (Kraków) 41(3–4): 65–75.
42. Kuznetsova VG, Maryańska-Nadachowska A, Glowacka E, da Silva PG (1995) Karyotypes of ten species of Psylloidea (Insecta: Homoptera) and some karyotaxonomical remarks. Beiträge zur Entomologie 45(2): 96–108.
43. Park H-Ch, Hodkinson ID, Kuznetsova VG (1995) Karyotypes of psyllid species (Homoptera: Psylloidea). Korean Journal of Entomology 25(2): 155–160. [In Korean with English summary].
44. Ovanesyan IG, Kuznetsova VG (1995) The karyotype of *Hydra vulgaris* Pall. and the survey of the karyotype data on other Hydridae species (Cnidaria, Hydrozoa, Hydroidea, Hydridae). In: Cnidaria. Modern and perspective investigations. II. Proceedings of the Zoological Institute, St. Petersburg, 261: 95–102. [In Russian with English summary].
45. Glowacka E, Kuznetsova VG, Maryańska-Nadachowska A (1995) Testis follicle number in psyllids (Psylloidea, Homoptera) as an anatomical feature in studies of systematic relations within the group. Folia Biologica (Kraków) 43(3–4): 115–124.
46. Kuznetsova VG, Maryańska-Nadachowska A (1996) Karyotypes of psyllids (Homoptera, Psylloidea), their evolution and taxonomic significance. In: Karyosystematics of the invertebrate animals III, Moscow, Botanical Garden, Moscow State University Publishing, 44–47. [In Russian with English summary].
47. Kuznetsova VG, Maryańska-Nadachowska A, Nokkala S (1996) Constitutive heterochromatin distribution in the karyotype of psyllid males of *Aphalara calthae* (L.) (Homoptera, Psylloidea). In: Karyosystematics of the invertebrate animals III, Moscow, Botanical Garden, Moscow State University Publishing, 47–49. [In Russian with English summary].
48. Golub NV, Grozeva SM, Kuznetsova VG (1996) Karyotypes of Psocoptera: a review and new data. In: Karyosystematics of the invertebrate animals III, Botanical Garden, Moscow State University Publishing, 20–22. [In Russian with English summary].
49. Maryańska-Nadachowska A, Kuznetsova VG, Yang Ch-T, Woudstra IH (1996) New data on karyotypes and the number of testicular follicles in the psyllid families Aphalaridae, Psyllidae, Carsidaridae and Triozidae (Homoptera, Psylloidea). Caryologia 49(3–4): 279–285. https://doi.org/10.1080/00087114.1996.10797372
50. Macharashvili ID, Kuznetsova VG (1997) Karyotypes, spermatogenesis, and morphology of the internal reproductive system in males of some psyllid species (Homoptera, Psylloidea) of the fauna of Georgia: I. Karyotypes and spermatogonial meiosis. Entomological Review 77(1): 12–20.
51. Kuznetsova VG, Nokkala S, Maryańska-Nadachowska A, Macharashvili ID (1997) Karyotypes, spermatogenesis, and morphology of the internal reproductive system in males of some psyllid species (Homoptera, Psylloidea) of the fauna from Georgia: II. Peculiarities of the reproductive system and initial stages of spermiogenesis. Entomological Review 77(1): 21–30.
52. Kuznetsova VG, Nokkala S, Maryańska-Nadachowska A (1997) Karyotypes, sex chromosome systems, and male meiosis in Finnish psyllids (Homoptera: Psylloidea). Folia Biologica (Kraków) 45(3–4): 143–152.
53. Kuznetsova VG, Maryańska-Nadachowska A, Nokkala S (1997) C-banded karyotype of psyllid species *Aphalara calthae* (L.) (Psylloidea, Homoptera, Insecta). Cytologia 62: 237–239. https://doi.org/10.1508/cytologia.62.237
54. Anokhin BA, Stepanjants SD, Kuznetsova VG (1998) Hydra fauna of Leningrad region and adjacent territory: taxonomy with the karyological analisys. Proceedings of the Zoological Institute RAS, 276: 19–26.
55. Shaposhnikov G, Kuznetsova V, Stekolshchikov A (1998) Evolutionary tendencies and system of Aphididae (Homoptera). In: Nieto Nafria JM, Dixon AFG (Eds) Aphids in Natural and Managed Ecosystems. Spain, León, 481–487.
56. Kuznetsova VG, Maryańska-Nadachowska A, Yang Ch-Tu, O’Brien L (1998) Karyotypes, sex-chromosome systems and testis structure in Fulgoroidea (Auchenorrhyncha, Homoptera, Insecta). Folia biologica (Kraków) 46(1–2): 23–40.
57. Stepanjants SD, Kuznetsova VG, Anokhin BA (1999) The fifth memoir about a freshwater polyp with horn-shaped arms. Priroda 7: 78–85. [In Russian].
58. Stepanjants SD, Kuznetsova VG, Anokhin BA (1999) A freshwater polyp with horn-shaped arms. “The fifth memoir”. In: Skulachev VP (Ed) Russian Science: The Present and the Future. Moscow: “Academia”, 245–254. [In Russian]
59. Westendorff M, Kuznetsova VG, Taeger A, Blank SM (1999) Karyotype diversity in the sawfly family Tenthredinidae (Symphyta, Hymenoptera): new data and review. Cytologia 64: 401–409. https://doi.org/10.1508/cytologia.64.401
60. Anokhin BA, Kuznetsova VG (1999) Chromosome morphology and banding patterns in *Hydra oligactis* Pallas and *H. circumcinta* Schultze (Hydroidea, Hydrida). Folia Biologica (Kraków), 47(3–4): 91–96.
61. Karagyan GA, Kuznetsova VG (2000) Chromosome numbers and sex chromosome systems in buprestid beetles (Coleoptera, Buprestidae). Entomological Review 80(1): 38–49.
62. Nokkala S, Kuznetsova V, Maryańska-Nadachowska A (2000) Achiasmate segregation of a B chromosome from the X chromosome in two species of psyllids (Psylloidea, Homoptera). Genetica (The Netherlands) 108: 181–189. https://doi.org/10.1023/a:1004146118610
63. Kuznetsova VG, Maryańska-Nadachowska A (2000). Autosomal polyploidy and male meiotic pattern in the bug family Nabidae (Heteroptera). Journal of Zoological Systematics and Evolutionary Research 38(2): 87–94. https://doi.org/10.1046/j.1439-0469.2000.382131.x
64. Stepanjants SD, Anokhin BA, Kuznetsova VG (2000) Hydrida composition and place in the system of Hydroidea (Cnidaria: Hydrozoa). Proceedings of Zoological Institute, Russian Academy of Sciences, St.Petersburg, 286: 155–162.
65. Alimov AF, Andriyashev AP, Borkin LYa, Darevskiy IS, Kerzhner IM, Kuznetsova VG, Starobogatov YaI, Stepanjants SD, Strelkov PP, Tanasijchuk VN, Khlebovich VV (2000) In memory of Nikolai N. Vorontsov (1934–2000). Zoologicheskii Zhurnal 79(11): 1369–1376. [In Russian].
66. Emeljanov AF, Golub NV, Kuznetsova VG (2001) Evolutionary transformation of testes and ovaries in booklice, birdlice, and sucking lice (Psocoptera, Phthiraptera: Mallophaga, Anoplura). Entomological Review 81(7): 767–785.
67. Kuznetsova VG, Westendorff M, Nokkala S (2001) Patterns of the chromosome banding in the sawfly family Tenthredinidae (Hymenoptera, Symphyta). Caryologia 54(3): 227–233. https://doi.org/10.1080/00087114.2001.10589230
68. Maryańska-Nadachowska A, Taylor GS, Kuznetsova VG (2001) Meiotic karyotypes and structure of testes in males of 17 species of Psyllidae: Spondyliaspidinae (Hemiptera: Psylloidea) from Australia. Australian Journal of Entomology 40: 349–356. https://doi.org/10.1046/j.1440-6055.2001.00230.x
69. Maryańska-Nadachowska A, Kuznetsova VG, Taylor GS (2001) Meiotic karyotypes and structure of testes in males of 12 species of Psyllidae: Acizzinae, Carsidaridae and Triozidae (Hemiptera: Psylloidea) from Australia. Australian Journal of Entomology 40: 357–364. https://doi.org/10.1046/j.1440-6055.2001.00231.x
70. Maryańska-Nadachowska A, Kuznetsova VG, Nokkala S (2001). Standard and C-banded meiotic karyotypes of Psylloidea (Sternorrhyncha, Homoptera, Insecta). Folia Biologica (Kraków) 49(1–2): 53–62.
71. Emeljanov AF, Golub NV, Kuznetsova VG (2001) Diversity and trends of the evolution of testes in Psocidea. Proceedings of the Zoological Institute, Russian Academy of Sciences 289: 67–74.
72. Kuznetsova VG, Stepanjants SD, Anokhin BA (2001) Karyosystematics – a new approach in study of Cnidaria. In: Evolution, ecology, and biodiversity. Proceedings of the Conference in memory of N.N. Vorontsov (1934–2000), Moscow State University Publishing, 119–130. [In Russian].
73. Borkin LYa, Alimov AF, Andriyashev AP, Darevskiy IS, Kerzhner IM, Kuznetsova VG, Starobogatov YaI, Stepanjants SD, Strelkov PP, Tanasijchuk VN, Khlebovich VV (2001). To the memory of N.N. Vorontsov (1934–2000). In: Evolution, ecology, and biodiversity. Proceedings of the Conference in memory of N.N. Vorontsov (1934–2000). Moscow State University Publishing, 8–21. [In Russian].
74. Kuznetsova VG, Nokkala S, Shcherbakov D (2002) Karyotype, reproductive organs, and pattern of gametogenesis in *Zorotypus hubbardi* Caudell (Insecta: Zoraptera, Zorotypidae), with discussion on relationships of the order. Canadian Journal of Zoology 80: 1047–1054. https://doi.org/10.1139/z02-074
75. Kuznetsova VG (2002) Karyosystematics. Priroda 8: 35–37. [In Russian].
76. Stepanjants SD, Kuznetsova VG, Anokhin BA (2003) Hydra: from Abraham Trembley to our time. ”Diversity of Animals” series. Issue 1. Moscow-St.Petersburg, 102 pp.
77. Medvedev GS, Bogdanova EN, Kipyatkov VE, Knyazev AN, Krivokhatskiy VA, Kuznetsova VG, Medvedev SG, Mikhailov KG, Narchuk EP, Pesenko YaA, Reznik SYa, Selikhovkin AV, Semjanov VP, Sinev SYu, Tobias VI (2003) XII Congress of Russian Entomological Society, St.Petersburg, August 19–24, 2002. Entomologicheskoe Obozrenie 82(1): 231–246. [In Russian].
78. Nokkala S, Grozeva S, Kuznetsova VG, Maryańska-Nadachowska A (2003) The origin of the achiasmatic XY sex chromosome system in *Cacopsylla peregrina* (Frst.) (Psylloidea, Homoptera). Genetica (Nederlands) 119: 327–332. https://doi.org/10.1023/b:gene.0000003757.27521.4d.
79. Kuznetsova VG, Maryańska-Nadachowska A, Nokkala S (2003) A new approach to the Auchenorrhyncha (Hemiptera, Insecta) cytogenetics: chromosomes of the meadow spittlebug *Philaenus spumarius* (L.) examined using various chromosome banding techniques. Folia Biologica (Kraków) 51(1–2): 33–40.
80. Kuznetsova VG (2003) Preface to: A.I. Shatalkin “Regulatory genes in development and a problem of morphotype in insect systematics”. Readings in memory of N.A. Kholodkovskij 56(2): 3–5.
81. Nechaeva GA, Kuznetsova VG, Nokkala S (2004) New data on the karyotype of *Pseudococcus viburni* (Sign.) (Homoptera, Coccinea). Entomologial Review 84(4): 393–400.
82. Kuznetsova VG, Golub NV (2004) Role of polyploidy in evolution of insects with holokinetic chromosomes. Proceedings of scientific studies of the St. Petersburg Scientific Centre. St. Petersburg University Publishing, 47–49. [In Russian].
83. Karagyan G, Kuznetsova VG, Lachowska D (2004) New cytogenetic data on Armenian buprestids (Coleoptera, Buprestidae) with a discussion of karyotype variation within the family. Folia biologica (Kraków) 52(3–4): 151–158. https://doi.org/10.3409/1734916044527601
84. Grozeva S, Kuznetsova VG, Nokkala S (2004) Patterns of chromosome banding in four nabid species (Heteroptera, Cimicomorpha, Nabidae) with high chromosome number karyotypes. Hereditas 140: 99–104. https://doi.org/10.1111/j.1601-5223.2004.01782.x
85. Golub NV, Nokkala S, Kuznetsova VG (2004) Holocentric chromosomes of psocids (Insecta, Psocoptera) analysed by C-banding, silver impregnation and sequence specific fluorochromes CMA3 and DAPI. Folia biologica (Kraków) 52(3–4): 143–149. https://doi.org/10.3409/1734916044527476
86. Kuznetsova V, Grozeva S, Nokkala S (2004) New cytogenetic data on Nabidae (Heteroptera: Cimicomorpha), with a discussion of karyotype variation and meiotic patterns, and their taxonomic significance. European Journal of Entomology 101: 205–210. https://doi.org/10.14411/eje.2004.026
87. Nokkala S, Kuznetsova V, Maryańska-Nadachowska A (2004) Holocentric chromosomes in meiosis. I. Restriction of the number of chiasmata in bivalents. Chromosome Research 12: 733–739. https://doi.org/10.1023/B:CHRO.0000045797.74375.70
88. Kerzhner IM, Kuznetsova VG, Rider DA (2004) Karyotypes of Pentatomoidea additional to those published by Ueshima, 1979 (Heteroptera). Zoosystematica Rossica 13(1): 17–21. https://doi.org/10.31610/zsr/2004.13.1.17
89. Gavrilov IA, Kuznetsova VG (2005) New data on the scale insect (Homoptera: Coccinea) fauna of European Russia, their taxonomy and cytogenetics (19–34 pp.). Proceedings of the X International Symposium on Scale Insect Studies 19–23 April 2004. Adana, 408 pp.
90. Kuznetsova VG, Gavrilov IA (2005) Scale insect cytogenetics (Insecta: Homoptera: Coccinea) – “terra incognita”? In: Stegniy VN (Ed) Evolutionary Biology, Proceedings of the III International Conference “Species and Speciation”, Tomsk, 20–22 October, 2004. Tomsk: Tomsk State University 3: 144–180. [In Russian].
91. Grozeva S, Kuznetsova VG, Nokkala S (2005) Cytogenetic study of the family Nabidae (Insecta, Heteroptera). In: Gruev B, Nikolova M, Donev A (Eds) Proceedings of the Balkan Scientific Conference of Biology in Plovdiv (Bulgaria), 19–21 of May 2005, Sofia, 553–559.
92. Kuznetsova VG, Golub NV, Gavrilov IA (2005). The dynamics of chromosome variability and peculiarities of meiosis in insects with holokinetic chromosomes. Proceedings of the reporting conference on the program of the Presidium of the Russian Academy of Sciences “Dynamics of plant, animal and human gene pools”, Moscow, 2005, 37–38. [In Russian].
93. Gokhman VE, Kuznetsova VG (2006) Comparative insect karyology: current state and applications. Entomological Review 86(3): 352–368. https://doi.org/10.1134/S0013873806030110
94. Aguin-Pombo D, Kuznetsova V, Freitas N (2006) Multiple parthenoforms of *Empoasca* leafhoppers from Madeira Island: where are these unisexual forms coming from? Journal of Heredity 97(2): 171–176. https://doi.org/10.1093/jhered/esj021
95. Nokkala S, Kuznetsova VG, Maryańska-Nadachowska A, Nokkala C (2006) Holocentric chromosomes in meiosis. II. The modes of orientation and segregation of a trivalent. Chromosome Research 14: 559–565. https://doi.org/10.1007/s10577-006-1053-6
96. Maryańska-Nadachowska A, Kuznetsova VG, Gnezdilov VM, Drosopoulos S (2006) Variability in the karyotypes, testes and ovaries of planthoppers of the families Issidae, Caliscelidae, and Acanaloniidae (Hemiptera: Fulgoroidea). European Journal of Entomology 103: 505–513. https://doi.org/10.14411/eje.2006.066
97. Stepanjants SD, Anokhin BA, Kuznetsova VG (2006) Cnidarian fauna of relict lakes Baikal, Biwa and Khubsugul. Hydrobiologia 568(S): 225–232. https://doi.org/10.1007/s10750-006-0310-1
98. Kuznetsova VG, Maryańska-Nadachowska A (2006) The male reproductive organs and karyotype of *Oeclidius* pr. *nanus* Van Duzee: first record for the family Kinnaridae (Homoptera: Fulgoroidea). Russian Entomological Journal 15(3): 281–286.
99. Labina ES, Maryańska-Nadachowska A, Kuznetsova VG (2007) Meiotic karyotypes in males of nineteen species of Psylloidea (Hemiptera) in the families Psyllidae and Triozidae. Folia biologica (Kraków) 55(1–2): 27–34. https://doi.org/10.3409/173491607780006353
100. Kuznetsova VG, Grozeva S, Sewlal Jo-AnneN, Nokkala S (2007). Cytogenetic characterization of the Trinidad endemic, *Arachnocoris trinitatus* Bergroth: the first data for the tribe Arachnocorini (Heteroptera: Cimicomorpha: Nabidae). Folia Biologica (Kraków) 55(1–2): 17–26. https://doi.org/10.3409/173491607780006344
101. Aguin-Pombo D, Franquinho Aguiar AM, Kuznetsova VG (2007) Bionomics and taxonomy of leafhopper *Sophonia orientalis* (Homoptera: Cicadellidae), a Pacific pest species in the Macaronesian Archipelagos. Annals of Entomological Society of America 100(1): 19–26. https://doi.org/10.1603/0013-8746(2007)100[19:BATOLS]2.0.CO;2
102. Nokkala C, Kuznetsova V, Grozeva S, Nokkala S (2007) Direction of karyotype evolution in the bug family Nabidae (Heteroptera): New evidence from 18S rDNA analysis. European Journal of Entomology 104: 661–665. https://doi.org/10.14411/eje.2007.083
103. Kuznetsova VG (2007) In Memoriam. Lydia Archipovna Chubareva. Comparative Cytogenetics 1(1): 95–96.
104. Gavrilov IA, Kuznetsova VG (2007) On some terms in scale cytogenetics and reproductive biology. Comparative Cytogenetics 1(2): 169–174.
105. Kuznetsova VG (2007) About the work of the karyosystematics, molecular systematics and insect genetics section at the XIII Congress of the All-Russian Entomological Society (Krasnodar, September 9–15, 2007). Comparative Cytogenetics 1(2): 176–178.
106. Grozeva S, Kuznetsova VG (2007) Karyotypes of the true bugs (Insecta, Heteroptera) – peculiarities, evolution and taxonomic significance. In: Evolution and Ecology – 2007, Proceedings, Sofia, 67–69.
107. Kuznetsova VG, Golub NV, Labina ES, Nechaeva GA, Gavrilov IA (2007) The dynamics of karyotype variability in closely related species from different Paraneopteran orders. Proceedings of the Program of basic research of the Presidium of Russian Academy of Sciences “Biodiversity and the dynamics of gene pools”, Moscow, 2007, 8–11. [In Russian].
108. Kuznetsova VG, Anokhin BA, Golub NV, Nechaeva GA, Petrova NA, Labina ES, Gavrilov IA (2007) Using of cytogenetic and molecular markers for studying of the gene pools dynamics of Insecta and Cnidaria. Proceedings of the reporting conference on the Program of basic research of the Presidium of Russian Academy of Sciences “Biodiversity and the dynamics of gene pools”, Moscow, 2007, 25–28. [In Russian].
109. Maryańska-Nadachowska A, Kuznetsova VG, Abdul-Nour H (2008) A chromosomal study on a Lebanese spittlebug *Philaenus arslani* (Hemiptera: Auchenorrhyncha: Aphrophoridae). European Journal of Entomology 105(2): 205–210. https://doi.org/10.14411/eje.2008.025
110. Drosopoulos S, Maryańska-Nadachowska A, Kuznetsova VG (2008) Additional new data proving that early publications on the polymorphism of the spittlebug of the genus *Philaenus* were lacking in systematics. Bulletin of Insectology 61(1): 99–100.
111. Maryańska-Nadachowska A, Kuznetsova VG, Drosopoulos S, Lachowska-Cierlik D (2008) A chromosomal analysis of eight Mediterranean species of *Philaenus*. Bulletin of Insectology 61(1): 133–134.
112. Maryańska-Nadachowska A, Lachowska-Cierlik D, Drosopoulos S, Kajtoch L, Kuznetsova VG (2008) A preliminary genetic study of Mediterranean species of *Philaenus* based on COI and ITS2 DNA sequences. Bulletin of Insectology 61(1): 135–136.
113. Nokkala S, Maryańska-Nadachowska A, Kuznetsova VG (2008) First evidence of polyploidy in Psylloidea (Homoptera, Sternorrhyncha): a parthenogenetic population of *Cacopsylla myrtilli* (W. Wagner, 1947) from northeast Finland is apomictic and triploid. Genetica 133: 201–205. https://doi.org/10.1007/s10709-007-9200-3
114. Kuznetsova VG, Grozeva S (2008) Cytogenetic characters of *Arachnocoris trinitatus* Bergroth, 1916 (Insecta: Heteroptera: Nabidae) from nests of the spider *Coryssocnemis simla* Huber, 2000 (Araneae: Pholcidae). Comparative Cytogenetics 2(2): 139–142.
115. Lukhtanov VA, Kuznetsova VG (2009) Molecular and cytogenetic approaches to species diagnostics, systematics, and phylogenetics. Zhurnal Obshchej Biologii 70(5): 415–437. [In Russian].
116. Kuznetsova VG, Maryańska-Nadachowska A, Emeljanov AF (2009) A contribution to the karyosystematics of the planthopper families Dictyopharidae and Fulgoridae (Hemiptera: Auchenorrhyncha). European Journal of Entomology 106: 159–170. https://doi.org/10.14411/eje.2009.019
117. Kuznetsova VG, Maryańska-Nadachowska A, Nokkala S (2009) Karyotype characterization of planthopper species *Hysteropterum albaceticum* Dlabola, 1983 and *Agalmatium bilobum* (Fieber, 1877) (Homoptera: Auchenorrhyncha: Issidae) using AgNOR-, C- and DAPI/CMA _3_- banding techniques. Comparative Cytogenetics 3(2): 111–123. https://doi.org/10.3897/compcytogen.v3i2.18
118. Labina ES, Nokkala S, Maryańska-Nadachowska A, Kuznetsova VG (2009) The distribution and population sex ratio of *Cacopsylla myrtilli* (W. Wagner, 1947) (Hemiptera: Psylloidea). Folia biologica (Kraków) 57(3–4): 157–163. https://doi.org/10.3409/fb57_3-4.157-163
119. Strelkov PP, Kuznetsova VG (2009) The entomologist I.M. Kerzhner. Priroda 6: 58–68. [In Russian].
120. Drosopoulos S, Maryańska-Nadachowska A, Kuznetsova VG (2010) The Mediterranean: area of origin of polymorphism and speciation in the spittlebug *Philaenus* (Hemiptera, Aphrophoridae). Zoosystematics and Evolution 86(1): 125–128. https://doi.org/10.1002/zoos.200900017
121. Kuznetsova VG, Grozeva S (2010) Achiasmatic meiosis: a review. The Herald of Vavilov Society for Geneticists and Breeding Scientists 14(1): 79–88. [In Russian]
122. Maryańska-Nadachowska A, Drosopoulos S, Lachowska D, Kajtoch Ł, Kuznetsova VG (2010) Molecular phylogeny of the Mediterranean species of *Philaenus* (Hemiptera: Auchenorrhyncha: Aphrophoridae) using mitochondrial and nuclear DNA sequences. Systematic Entomology 35(1): 318–328. https://doi.org/10.1111/j.1365-3113.2009.00510.x
123. Kuznetsova VG, Maryańska-Nadachowska A, Gnezdilov VM (2010) Meiotic karyotypes and testis structure in 14 species of the planthopper tribe Issini (Hemiptera: Fulgoroidea, Issidae). European Journal of Entomology 107: 465–480. https://doi.org/10.14411/eje.2010.055
124. Lukhtanov VA, Kuznetsova VG (2010) What genes and chromosomes say about the origin and evolution of insects and other arthropods. Russian Journal of Genetics 46(9): 1115–1121. https://doi.org/10.1134/S1022795410090279
125. Grozeva S, Kuznetsova V, Anokhin B (2010) Bed bug cytogenetics: karyotype, sex chromosome system, FISH mapping of 18S rDNA, and male meiosis in *Cimex lectularius* Linnaeus, 1758 (Heteroptera: Cimicidae). Comparative Cytogenetics 4(2): 151–160. https://doi.org/10.3897/compcytogen.v4i2.36
126. Grozeva S, Kuznetsova VG, Anokhin BA (2011) Karyotypes, male meiosis and comparative FISH mapping of 18S ribosomal DNA and telomeric (TTAGG)n repeat in eight species of true bugs (Hemiptera, Heteroptera). Comparative Cytogenetics 5(4): 355–374. https://doi.org/10.3897/CompCytogen.v5i4.2307
127. Kuznetsova VG, Grozeva S, Nokkala S, Nokkala C (2011) Cytogenetics of the true bug infraorder Cimicomorpha (Hemiptera: Heteroptera): a review. ZooKeys 154: 31–70. https://doi.org/10.3897/zookeys.154.1953
128. Kuznetsova VG, Grozeva SM, Anokhin BA (2012) The first finding of (TTAGG)n telomeric repeat in chromosomes of true bugs (Heteroptera: Belostomatidae: *Lethocerus patruelis*). Comparative Cytogenetics 6(4): 341–346. https://doi.org/10.3897/CompCytogen.v6i4.4058
129. Maryańska-Nadachowska A, Kuznetsova VG, Lachowska D, Drosopoulos S (2012) Mediterranean species of the spittlebug genus *Philaenus*: Modes of chromosome evolution. Journal of Insect Science 12:54. https://doi.org/10.1673/031.012.5401
130. Kuznetsova VG, Labina ES, Shapoval NA, Maryańska-Nadachowska A, Lukhtanov VA (2012) *Cacopsylla fraudatrix* sp.n. (Hemiptera: Psylloidea) recognized from testis structure and mitochondrial gene COI. Zootaxa 3547: 55–63. https://doi.org/10.11646/zootaxa.3547.1.5
131. Grozeva S, Kuznetsova V, Simov N, Langurov M, Dalakchieva S (2013) Sex chromosome “pre-reduction” in male meiosis of *Lethocerus patruelis* (Stål, 1854) (Heteroptera, Belostomatidae) with some notes on the distribution of the species. ZooKeys 319: 119–135. https://doi.org/10.3897/zookeys.319.4384
132. Nokkala C, Kuznetsova VG, Nokkala S (2013) Meiosis in rare males in parthenogenetic *Cacopsylla myrtilli* (Wagner, 1947) (Hemiptera, Psyllidae) populations from northern Europe. Comparative Cytogenetics 7(3): 241–251. https://doi.org/10.3897/CompCytogen.v7i3.6126
133. Maryańska-Nadachowska A, Kuznetsova VG, Karamysheva TV (2013) Chromosomal location of rDNA clusters and TTAGG telomeric repeats in eight species of the spittlebug genus *Philaenus* (Hemiptera: Auchenorrhyncha: Aphrophoridae). European Journal Entomology 110(3): 411–418. https://doi.org/10.14411/eje.2013.055
134. Kuznetsova VG, Golub NV, Aguin-Pombo D (2013) Karyotypes, B-chromosomes and meiotic abnormalities in 13 populations of *Alebra albostriella* and *A. wahlbergi* (Hemiptera, Auchenorrhyncha, Cicadellidae) from Greece. Comparative Cytogenetics 7(4): 305–325. https://doi.org/10.3897/CompCytogen.v7i4.6411
135. Labina ES, Maryańska-Nadachowska A, Burckhardt D, Kuznetsova VG (2014) Variation in sperm formation patterns in jumping plant-lice (Hemiptera: Psylloidea): a light microscopic study. Folia biologica (Kraków) 62(4): 321–333. https://doi.org/10.3409/fb62_4.321
136. Grozeva S, Kuznetsova V, Hartung V (2014) First cytogenetic study of Coleorrhyncha: meiotic complement of *Xenophyes cascus* (Hemiptera: Peloridiidae). European Journal of Entomology 111(2): 303–306. https://doi.org/10.14411/eje.2014.023
137. Gokhman VE, Anokhin BA, Kuznetsova VG (2014) Distribution of 18S rDNA sites and absence of the canonical TTAGG insect telomeric repeat in parasitoid Hymenoptera. Genetica 142: 317–322. https://doi.org/10.1007/s10709-014-9776-3
138. Golub NV, Kuznetsova VG, Rakitov RA (2014) First karyotype data on the family Myerslopiidae (Hemiptera, Auchenorrhyncha, Cicadomorpha). Comparative Cytogenetics 8(4): 293–300. https://doi.org/10.3897/CompCytogen.v8i4.8813
139. Grozeva S, Anokhin BA, Kuznetsova VG (2015) Bedbugs (Hemiptera). In: Sharakhov I (Ed) Protocols for cytogenetic mapping of arthropod genomes. CRC Press, Taylor and Francias Group, London, New York, 285–326. https://doi.org/10.1201/b17450-9
140. Kuznetsova VG, Maryańska-Nadachowska A, Karamysheva T (2015) Spittlebugs (Hemiptera). In: Sharakhov I (Ed) Protocols for cytogenetic mapping of arthropod genomes. CRC Press, Taylor and Francias Group, London, New York, 351–380. https://doi.org/10.1201/b17450-11
141. Kuznetsova VG, Grozeva SM, Hartung V, Anokhin BA (2015) First evidence for (TTAGG)n telomeric sequence and sex chromosome post-reduction in Coleorrhyncha (Insecta, Hemiptera). Comparative Cytogenetics 9(4): 523–532. https://doi.org/10.3897/CompCytogen.v9i4.5609
142. Golub NV, Golub VB, Kuznetsova VG (2015) Variability of 18rDNA loci in four lace bug species (Hemiptera, Tingidae) with the same chromosome number. Comparative Cytogenetics 9(4): 513–522 https://doi.org/10.3897/CompCytogen.v9i4.5376
143. Nokkala C, Kuznetsova VG, Nokkala S (2015) Rare diploid females coexist with rare males: a novel finding in triploid parthenogenetic populations in the psyllid *Cacopsylla myrtilli* (W. Wagner, 1947) (Hemiptera, Psylloidea) in northern Europe. Genetica 143: 589–595. https://doi.org/10.1007/s10709-015-9858-x
144. Kuznetsova VG, Maryańska-Nadachowska A, Anokhin BA, Aguin-Pombo D (2015) Evidence for TTAGG telomere repeats and rRNA gene clusters in leafhoppers of the genus *Alebra* (hemiptera: Auchenorrhyncha: Cicadellidae). European Journal of Entomology 112(2): 207–214. https://doi.org/10.14411/eje.2015.045
145. Kuznetsova VG, Khabiev GN, Krivokhatsky VA (2015) Chromosome numbers in antlions (Myrmeleontidae) and owlflies (Ascalaphidae) (Insecta, Neuroptera). ZooKeys 538: 47–61 https://doi.org/10.3897/zookeys.538.6655
146. Kuznetsova V, Aguin-Pombo D (2015) Comparative cytogenetics of Auchenorrhyncha (Hemiptera: Homopetera): a Review. ZooKeys 538: 63–93. https://doi.org/10.3897/zookeys.538.6724
147. Stoianova D, Grozeva S, Simov N, Kuznetsova V (2015) Achiasmate male meiosis in two *Cymatia* species (Hemiptera, Heteroptera, Corixidae). ZooKeys 538: 95–104. https://doi.org/10.3897/zookeys.538.6722
148. Lachowska-Cierlik D, Maryańska-Nadachowska A, Kuznetsova V, Picker M (2015) First chromosomal study of Mantophasmatodea: karyotype of *Karoophasma biedouwense* (Austrophasmatidae). European Journal of Entomology 112(4): 599–605. https://doi.org/10.14411/eje.2015.093
149. Lukhtanov VA, Shapoval NA, Anokhin BA, Saifitdinova AF, Kuznetsova VG (2015) Homoploid hybrid speciation and genome evolution via chromosome sorting. Proceedings of the Royal Society B 282: 20150157. https://doi.org/10.1098/rspb.2015.0157
150. Vershinina AO, Kuznetsova VG (2016) Parthenogenesis in Hexapoda: Entognatha and non-holometabolous insects. Journal of Zoological Systematics and Evolutionary Research 54(4): 257–268.
151. Maryańska-Nadachowska A, Anokhin BA, Gnezdilov VM, Kuznetsova VG (2016) Karyotype stability in the family Issidae (Hemiptera, Auchenorrhyncha) revealed by chromosome techniques and FISH with telomeric (TTAGG)n and 18S rDNA probes. Comparative Cytogenetics 10(3): 347–369. https://doi.org/10.3897/CompCytogen.v10i3.9672
152. Golub NV, Golub VB, Kuznetsova VG (2016) Further evidence for the variability of the 18S rDNA loci in the family Tingidae (Hemiptera, Heteroptera). Comparative Cytogenetics 10 (4): 517–527. https://doi.org/10.3897/CompCytogen.v10i4.9631
153. Kuznetsova VG, Khabiev GN, Anokhin BA (2016) Cytogenetic study on ant-lions (Neuroptera: Myrmeleontidae): first data on telomere structure and rDNA location. Comparative Cytogenetics 10(4): 647–656. https://doi.org/10.3897/CompCytogen.v10i4.10775
154. Stoianova D, Grozeva S, Simov N, Kuznetsova V (2017) Karyotype, sex determination and male meiosis in three benthic water bugs (Hemiptera: Nepomorpha: Aphelocheiridae). Aquatic Insects 38(3): 115–124. https://doi.org/10.1080/01650424.2017.1346260
155. Angus RB, Jeangirard C, Stoianova D, Grozeva S, Kuznetsova VG (2017) A chromosomal analysis of *Nepa cinerea* Linnaeus, 1758 and *Ranatra linearis* (Linnaeus, 1758) (Heteroptera, Nepidae). Comparative Cytogenetics 11(4): 641–657. https://doi.org/10.3897/CompCytogen.v11i4.14928
156. Golub NV, Golub VB, Kuznetsova VG (2017) Distribution of the major rDNA loci among four Hemipteran species of the family Tingidae (Heteroptera, Cimicomorpha). Folia biologica (Kraków) 65(3): 155–158. https://doi.org/10.3409/fb65_3.155
157. Nokkala S, Kuznetsova VG, Nokkala C (2017) Characteristics of parthenogenesis in *Cacopsylla ledi* (Flor, 1861) (Hemiptera, Sternorryncha, Psylloidea): cytological and molecular approaches. Comparative Cytogenetics 11(4): 807–817. https://doi.org/10.3897/CompCytogen.v11i4.21362
158. Kuznetsova V (2017) Book Review: Kiknadze I, Istomina A, Golygina V, Gunderina L. Karyotypes of Palearctic and Holarctic species of the genus *Chironomus* [Electronic resource] Russian Academy of Sciences, Siberian Branch, Federal Research Center, Institute of Cytology and Genetics. Comparative Cytogenetics 11(2): 431–433. https://doi.org/10.3897/CompCytogen.v11i2.13527
159. Kuznetsova VG, Maryańska-Nadachowska A, Shapova NA, Anokhin BA, Shapoval AP (2017) Cytogenetic characterization of eight Odonata species originating from the Curonian Spit (the Baltic Sea, Russia) using C-banding and FISH with 18S rDNA and telomeric (TTAGG)n probes. Cytogenetic and Genome Research 153(3): 147–157. https://doi.org/10.1159/000486088
160. Gokhman VE, Kuznetsova VG (2018) Parthenogenesis in Hexapoda: holometabolous insects. Journal of Zoological Systematics and Evolutionary Research 56(1): 23–34. https://doi.org/10.1111/jzs.12183
161. Gokhman VE, Kuznetsova VG (2018) Presence of the canonical TTAGG insect telomeric repeat in the Tenthredinidae (Symphyta) suggests its ancestral nature in the order Hymenoptera. Genetica 146(3): 341–344. https://doi.org/10.1007/s10709-018-0019-x
162. Gokhman VE, Kuznetsova VG (2018) Phylogenetic distribution of the canonical insect TTAGG telomeric repeat within the order Hymenoptera (Insecta). Comparative Cytogenetics 12(3): 324–325. https://doi.org/10.3897/CompCytogen.v12i3.27748
163. Maryańska-Nadachowska A, Kuznetsova VG, Golub NV, Anokhin BA (2018) Detection of telomeric sequences and ribosomal RNA genes in holokinetic chromosomes of five jumping plant-lice species: First data on the superfamily Psylloidea (Hemiptera: Sternorrhyncha). European Journal of Entomology 115: 632–640. https://doi.org/10.14411/eje.2018.061
164. Golub NV, Golub VB, Kuznetsova VG (2018) New data on karyotypes of lace bugs (Tingidae, Cimicomorpha, Hemiptera) with analysis of the 18S rDNA clusters distribution. Comparative Cytogenetics 12(4): 515–528. https://doi.org/10.3897/CompCytogen.v12i4.30431
165. Anokhin BA, Kuznetsova VG (2018) FISH-based karyotyping of *Pelmatohydra oligactis* (Pallas, 1766), *Hydra oxycnida* Schulze, 1914, and *H. magnipapillata* Ito, 1947 (Cnidaria, Hydrozoa, Hydrida, Hydridae). Comparative Cytogenetics 12(4): 539–548. https://doi.org/10.3897/CompCytogen.v12i4.32120
166. Michailova P, Kuznetsova VG, Grozeva S, Ilkova J, Petrova NA (2018) In Memoriam: Professor Iya Kiknadze (1930–2017). Comparative Cytogenetics 12(1): 141–144. https://doi.org/10.3897/CompCytogen.v12i1.24550
167. Kuznetsova V, Golub N, Petrova N, Lukhtanov V, Anokhin B, Khabasova N, Shapoval N, Kupriyanova L, Gavrilov-Zimin I (2019) In Memoriam: Dr. Sergey V. Zhirov (1966–2017). Comparative Cytogenetics 13(3): 321–324. https://doi.org/10.3897/CompCytogen.v13i3.47366
168. Nokkala C, Kuznetsova VG, Rinne V, Nokkala S (2019) Description of two new species of the genus *Cacopsylla* Ossiannilsson, 1970 (Hemiptera, Psylloidea) from northern Fennoscandia recognized by morphology, cytogenetic characters and COI barcode sequence. Comparative Cytogenetics 13(4): 367–382. https://doi.org/10.3897/CompCytogen.v13i4.47395
169. Grozeva S, Anokhin BA, Simov N, Kuznetsova VG (2019) New evidence for the presence of the telomere motif (TTAGG)n in the family Reduviidae and its absence in the families Nabidae and Miridae (Hemiptera, Cimicomorpha). Comparative Cytogenetics 13(3): 283–295. https://doi.org/10.3897/CompCytogen.v13i3.36676
170. Golub N, Anokhin B, Kuznetsova V (2019) Comparative FISH mapping of ribosomal DNA clusters and TTAGG telomeric sequences to holokinetic chromosomes of eight species of the insect order Psocoptera. Comparative Cytogenetics 13(4): 403–410. https://doi.org/10.3897/CompCytogen.v13i4.48891
171. Kuznetsova VG, Maryańska-Nadachowska A, Khabiev GN, Karagyan G, Krivokhatsky VA (2019) Variation in the number of testicular follicles and ovarioles among 18 lacewing species of the families Myrmeleontidae, Ascalaphidae, and Nemopteridae (Insecta, Neuroptera, Myrmeleontiformia). ZooKeys 894: 33–51. https://doi.org/10.3897/zookeys.894.47040
172. Kuznetsova VG, Grozeva S, Gokhman VE (2020) Telomere structure in insects: A review. Journal of Zoological Systematics and Evolutionary Research 58: 127–158. https://doi.org/10.1111/jzs.12332
173. Kuznetsova VG, Golub NV (2020) A checklist of chromosome numbers and a review of karyotype variation in Odonata of the world. Comparative Cytogenetics 14(4): 501–540. https://doi.org/10.3897/compcytogen.v14.i4.57062
174. Kuznetsova VG, Maryańska-Nadachowska A, Anokhin BA, Shapoval NA, Shapoval AP (2020) Chromosomal analysis of eight species of dragonflies (Anisoptera) and damselflies (Zygoptera) using conventional cytogenetics and fluorescence in situ hybridization: Insights into the karyotype evolution of the ancient insect order Odonata. Journal of Zoological Systematics and Evolutionary Research 58: 1–13. https://doi.org/10.1111/jzs.12429
175. Gapon DA, Kuznetsova VG, Maryańska-Nadachowska A (2021) A new species of the genus *Rhaphidosoma* Amyot et Serville, 1843 (Heteroptera, Reduviidae), with data on its chromosome complement. Comparative Cytogenetics 15(4): 467–505. https://doi.org/10.3897/compcytogen.v15.i4.78718
176. Gavrilov-Zimin IA, Grozeva SM, Gapon DA, Kurochkin AS, Trencheva KG, Kuznetsova VG (2021) Introduction to the study of chromosomal and reproductive patterns in Paraneoptera. Comparative Cytogenetics 15(3): 217–238. https://doi.org/10.3897/compcytogen.v15.i3.69718
177. Kuznetsova VG, Gavrilov-Zimin IA, Grozeva SM, Golub NV (2021) Comparative analysis of chromosome numbers and sex chromosome systems in Paraneoptera (Insecta). Comparative Cytogenetics 15(3): 279–327. https://doi.org/10.3897/compcytogen.v15.i3.71866
178. Shapoval NA, Nokkala S, Nokkala C, Kuftina GN, Kuznetsova VG (2021) The incidence of *Wolbachia* bacterial endosymbiont in bisexual and parthenogenetic populations of the psyllid genus *Cacopsylla* (Hemiptera, Psylloidea). Insects 12: 853. https://doi.org/10.3390/insects12100853
179. Nokkala C, Kuznetsova VG, Shapoval NA, Nokkala S (2022) Phylogeography and *Wolbachia* infections reveal postglacial recolonization routes of the parthenogenetic plant louse *Cacopsylla myrtilli* (W. Wagner 1947), (Hemiptera, Psylloidea). Journal of Zoological Systematics and Evolutionary Research, 5458633. https://doi.org/10.1155/2022/5458633
180. Gokhman VE, Kuznetsova VG (2022) Chapter 27 FISH – in Insect Chromosomes. In: Liehr T (Ed.) Cytogenetics and molecular genetics. CRC Press Taylor and Francis Group, London, 319–338. https://doi.org/10.1201/9781003223658-27
181. Gokhman VE, Kuznetsova VG, Sharakhov IV (2022) Editorial: Evolutionary cytogenetics of insects. Frontiers in Ecology and Evolution 10: 994136. https://doi.org/10.3389/fevo.2022.994136
182. Golub NV, Golub VB, Anokhin BA, Kuznetsova VG (2022) Comparative cytogenetics of lace bugs (Tingidae, Heteroptera): New data and a brief overview. Insects 13: 608. https://doi.org/10.3390/insects13070608
183. Nokkala S, Kuznetsova V, Pietarinen P, Nokkala C (2022) Evolutionary potential of parthenogenesis – bisexual lineages within triploid apomictic thelytoky in *Cacopsylla ledi* (Flor, 1861) (Hemiptera, Psylloidea) in Fennoscandia. Insects 13: 1140. https://doi.org/10.3390/insects13121140
184. Grozeva S, Stoianova D, Konstantinov F, Simov N, Kuznetsova VG (2022) A synopsis of the numbers of testicular follicles and ovarioles in true bugs (Heteroptera, Hemiptera) – sixty-five years of progress after J. Pendergrast’s review. ZooKeys 1136: 71–123. https://doi.org/10.3897/zookeys.1136.96431
185. Golub NV, Maryańska-Nadachowska A, Anokhin BA, Kuznetsova VG (2023) Expanding the chromosomal evolution understanding of lygaeioid true bugs (Lygaeoidea, Pentatomomorpha, Heteroptera) by classical and molecular cytogenetic analysis. Genes 14, 725. https://doi.org/10.3390/genes14030725
186. Golub NV, Anokhin BA, Kuznetsova VG (2023) Karyotype diversity in the genus *Nysius* Dallas, 1852 (Hemiptera, Heteroptera, Lygaeidae) is much greater than you might think. Comparative Cytogenetics 17: 287–293. https://doi.org/10.3897/compcytogen.17.116628
187. Aguín-Pombo D, Kuznetsova VG (2023) True parthenogenesis and female-biased sex ratios in Cicadomorpha and Fulgoromorpha (Hemiptera, Auchenorrhyncha). Insects 14: 820. https://doi.org/10.3390/insects14100820
188. Aguín-Pombo D, Kuznetsova VG (2023) Parthenogenesis in Fulgoromorpha and Cicadomorpha. Encyclopedia. Subjects: Environmental Sciences. https://encyclopedia.pub/entry/50874
189. Kuznetsova VG, Golub NV, Maryańska-Nadachowska A (2024) Number of seminal follicles and ovarioles in Fulgoromorpha (Hemiptera: Auchenorrhyncha): Variability and evolutionary trends. European Journal of Entomology 121: 109–123. https://doi.org/10.14411/eje.2024.014
190. Gokhman VE, Kuznetsova VG (2024) Structure and evolution of ribosomal genes of insect chromosomes. Insects 15: 593. https://doi.org/10.3390/insects15080593
191. Stoianova D, Grozeva S, Golub NV, Anokhin BA, Kuznetsova VG (2024) The first FISH confirmed non-canonical telomeric motif in Heteroptera: *Cimex lectularius* Linnaeus, 1758 and *C. hemipterus* (Fabricius, 1803) (Hemiptera, Cimicidae) have a 10 bp motif (TTAGGGATGG)n. Genes 15: 1026 https://doi.org/10.3390/genes15081026
192. Kuznetsova VG, Golub NV (2024) The number of testicular follicles and ovarioles in Cicadomorpha (Hemiptera: Auchenorrhyncha): Variability and evolutionary trends. European Journal of Entomology 121: 413–424. https://doi.org/10.14411/eje.2024.045
193. Kuznetsova V, Michailova P, Przhiboro A, Khabazova N (2024) In Memoriam: Cytogeneticist Dr. Sc. Ninel A. Petrova (1940–2024) – life and scientific heritage. Comparative Cytogenetics 18: 277–305. https://doi.org/10.3897/compcytogen.18.138747
194. Alekseev VR, Kuznetsova VG, Nartshuk EP (2024) In memoriam: Elena Borisovna Vinogradova, an outstanding scientist and a remarkable person (1933–2021). Entomologicheskoe Obozrenie 103(4): 418–434.
195. Gokhman VE, Kuznetsova VG, Anokhin BA (2024) Number and location of rDNA clusters in the superfamilies Tenthredinoidea and Cynipoidea (Hymenoptera): an update. Comparative Cytogenetics 18: 239–246. https://doi.org/10.3897/compcytogen.18.142301
196. Golub N, Anokhin B, Kuznetsova V (2025) Non-canonical telomeric motif TTAGGGGTGG in the true bug species *Geocoris dispar* Waga, 1839 (Heteroptera: Geocoridae). Comparative Cytogenetics 19: 117–123. https://doi.org/10.3897/compcytogen.19.156983
197. Kuznetsova V, Golub N, Anokhin B, Stoianova D, Lukhtanov V (2025) Diversity of telomeric sequences in true bugs (Heteroptera): new data on the infraorders Pentatomomorpha and Cimicomorpha. Cytogenetic and Genome Research 165 (3–5): 192–205. https://doi.org/10.1159/000545902
198. Stoianova D, Grozeva S, Todorova N, Rangelov M, Lukhtanov VA, Kuznetsova VG (2025) New insights into the telomere structure in Hemiptera (Insecta) inferred from chromosome-level and scaffold-level genome assemblies. Diversity 17: 552. https://doi.org/10.3390/d17080552
199. Shapoval NA, Nokkala S, Nokkala C, Shapoval GN, Labina ES, Romanovich AE, Kuznetsova VG (2025) Genetic differentiation of bisexual and parthenogenetic populations of plant louse *Cacopsylla ledi* (Hemiptera, Psylloidea). Insects 16(12): 1268. https://doi.org/10.3390/insects16121268
200. Golub NV, Anokhin BA, Grozeva S, Kuznetsova VG (2025) Comparative chromosomal mapping of the 18S rDNA loci in true bugs: the first data for 13 genera of the infraorders Cimicomorpha and Pentatomomorpha (Hemiptera, Heteroptera). Genes 18(16): 1516. https://doi.org/10.3390/genes16121516

